# Transcriptome Analysis of Responses to Rhodomyrtone in Methicillin-Resistant *Staphylococcus aureus*

**DOI:** 10.1371/journal.pone.0045744

**Published:** 2012-09-27

**Authors:** Wipawadee Sianglum, Potjanee Srimanote, Peter W. Taylor, Helena Rosado, Supayang P. Voravuthikunchai

**Affiliations:** 1 Department of Microbiology and Natural Products Research Center, Faculty of Science, Prince of Songkla University, Songkla, Thailand; 2 Graduate Study, Faculty of Allied Health Sciences, Thammasat University, Pathumtanee, Thailand; 3 School of Pharmacy, University College London, London, United Kingdom; National Institutes of Health, United States of America

## Abstract

Rhodomyrtone, purified from *Rhodomyrtus tomentosa* (Aiton) Hassk, exhibits a high degree of potency against methicillin-resistant *Staphylococcus aureus* (MRSA). We recently demonstrated that exposure of MRSA to a subinhibitory concentration (0.174 µg/ml) of rhodomyrtone resulted in the alteration of expression of several functional classes of bacterial proteins. To provide further insight into the antibacterial mode of action of this compound, we determined the impact of exposure to rhodomyrtone on the gene transcriptional profile of MRSA using microarray analysis. Exposure of MRSA to subinhibitory concentrations (0.5MIC; 0.5 µg/ml) of rhodomyrtone revealed significant modulation of gene expression, with induction of 64 genes and repression of 35 genes. Prominent changes in response to exposure to rhodomyrtone involved genes encoding proteins essential to metabolic pathways and processes such as amino acid metabolism, membrane function, ATP-binding cassette (ABC) transportation and lipoprotein and nucleotide metabolism. Genes involved in the synthesis of the aspartate family of amino acids, in particular proteins encoded by the *dap* operon were prominent. The diaminopimelate (DAP) biosynthetic pathway is the precursor of lysine synthesis and is essential for peptidoglycan biosynthesis. However, phenotypic analysis of the peptidoglycan amino acid content of rhodomyrtone-treated MRSA did not differ significantly from that extracted from control cells. Genes involved in the biosynthesis of amino acids and peptidoglycan, and a high affinity ATP-driven K (^+^) transport system, were investigated by quantitative reverse transcription-PCR (qRT-PCR) using EMRSA-16 1, 4, or 18 h after exposure to rhodomyrtone and in general the data concurred with that obtained by microarray, highlighting the relevance of the DAP biosynthetic pathway to the mode of action of rhodomyrtone.

## Introduction

Methicillin-resistant *Staphylococcus aureus* (MRSA) is a major cause of hospital- and community-acquired infections [1], [2] and continues to evolve with respect to the emergence of resistance to commonly used antibiotic classes [3]. Linezolid, quinupristin-dalfopristin, daptomycin, telavancin, new glycopeptides, and ceftobiprole are new antimicrobial agents which have been introduced or are under clinical development for the treatment of infections due to drug resistant bacteria [4], [5] but resistance to these agents is, in turn, emerging [6], [7], [8], [9]. Thus, there is a continuing need to discover and develop new therapeutic agents to combat these ememrging drug-resistant bacteria [10]. Natural bioactive compounds and medicinal plants have been successfully used in the treatment of several bacterial infections [11], [12], [13] and may continue to provide a source of much-needed new agents to replenish our dwindling antibacterial armamentarium.

*Rhodomyrtus tomentosa* (Aiton) Hassk. is an ornamental evergreen shrub of the family Myrtaceae. It is native to Southeast Asia and is a troublesome invader of native plant communities in Florida. Crude extracts from leaves of this plant possess good antibacterial efficacy against *Escherichia coli* and *S*. *aureus* [14] and at least part of this activity is attributable to rhodomyrtone, an acylphloroglucinol derivative [15]. The compound has been shown to possess significant antibacterial activity against several Gram-positive bacteria such as *Bacillus subtilis*, *Enterococcus faecalis*, *S. aureus*, *S. epidermidis*, *Streptococcus* spp., and MRSA [14], [16], [17], [18].

We have recently undertaken a proteomic analysis of MRSA challenged with subinhibitory concentrations of rhodomyrtone; the compound modulates expression of a number of proteins involved in cell wall biosynthesis, cell division, stress responses and cell surface antigen and virulence factor expression, as well as metabolic pathways mediating amino acid metabolism [18]. In addition, transmission electron micrographs of rhodomyrtone-treated MRSA revealed pronounced damage to the cell envelope and the septum in the abence of any perturbation of the amino acid content of the peptidoglycan-wall teichoic acid complex [18]. We were, however, unable to identify the primary target for rhodomyrtone within MRSA. Here, we report global transcriptional responses of EMRSA-16 after 1 h exposure to rhodomyrtone. Time course analysis of the bacterial transcriptome after exposure to the compound for 1, 4, and 18 h was undertaken by the quantitative real time-polymerase chain reaction (qRT-PCR). Genome expression profiling of MRSA with and without rhodomyrtone will provide an opportunity to gain insights into the mechanism of action of the compound.

## Results and Discussion

### Rhodomyrtone-induced Changes in Gene Expression

The global transcriptional response of MRSA to rhodomyrtone using a *S. aureus* microarray for the profiling of genes modulated by this agent. The minimum inhibitory concentration (MIC) and minimum bactericidal concentration (MBC) of rhodomyrtone against EMRSA-16 by broth microdilution were 1.0 µg/ml and 4.0 µg/ml, respectively. The microarray analysis was designed to investigate the effects of rhodomyrtone at 0.5MIC (0.5 µg/ml) on gene expression of EMRSA-16 after a short incubation period of 1 h. Transcript abundance from mid log-phase cells grown in MHB in the presence or absence of rhodomyrtone were compared by Affymetrix microarray analysis. Microarray data were further validated by quantitative real-time reverse transcriptase-polymerase chain reaction (qRT-PCR) analysis of selected genes. Statistically significant twofold or greater changes were demonstrated for 64 rhodomyrtone-induced genes and 35 rhodomyrtone-repressed genes (Tables S1 and S2). Up- and down-regulated genes were categorized into three groups: (i), <10 fold change, 61 genes (61.6%), (ii), 10–50 fold change, 22 genes (22.2%), and (iii) >50 fold change, 16 genes (16.2%). Genes up-regulated by growth in MHB containing rhodomyrtone included genes involved in the metabolism of amino acids, putative membrane proteins, transporter proteins, lipoproteins, exported proteins, nucleotide metabolism, virulence factors and cell wall metabolism.

Rhodomyrtone strongly up-regulated a group of genes involved in amino acid biosynthetic pathways; these included genes encoding proteins involved in lysine, threonine and arginine biosynthesis, such as aspartate semialdehyde dehydrogenase *(asd*), dihydrodipicolinate synthase (*dapA*), dihydrodipicolinate reductase (*dapB*), and tetrahydrodipicolinate (*dapD*),threonine synthase (*thrC*), and putative argininosuccinate synthase (*argG*). Diaminopimelate (DAP) biosynthetic pathway produces not only the precursors for methionine, homoserine, threonine, lysine biosynthesis, but also for the other amino acids such as arginine and proline, all of which are essential for cell cycle and metabolism. Therefore, it is possible that the await-to-be elucidated antibacterial mechanism of rhodomyrtone lies in there. Based on structure comparison, rhodomyrtone showed no similarities with DAP, any of its biosynthetic precursors, or intermediate products of methionine, homoserine, threonine, and lysine biosynthesis pathway. Therefore, rhodomyrtone is unlikely to act as amino acid analog for its antibacterial mechanism. However, whether it interferes with enzymes in these biosynthetic pathways is still under investigation.

DAP biosynthesis is an attractive target for the development of antibacterial drugs as it is essential for peptidoglycan biosynthesis and DAP is not required by humans [24], [25]. In this study, microarray data revealed an up-regulation of a group of genes in the DAP operon. The essential nature of bacterial DAP biosynthesis is determined by its absolute requirement as a component of peptidoglycan and its role as a direct precursor of lysine [26]. Thus, rhodomyrtone may impact on the generation of lysine and the structural integrity of peptidoglycan. Transcriptomic data also revealed an increase in transcription of the *tagG* genes responsible for teichoic acid biosynthesis [27].

The up-regulation of four ATP-binding cassette (ABC) transporters and related genes indicated potential modulation of genes involved in the export of toxins across the cytoplasmic membrane. Moreover, rhodomyrtone affected several genes encoding bacterial membrane proteins: seven (10.6%) genes were strongly induced whereas five (13.9%) genes were weakly down-regulated.

Two genes, including SAR2030 (encoding MHC class II analog) and *sbi* (encoding IgG-binding protein) involved in virulence determination, were up-regulated. Major histocompatibility complex (MHC) class II analog, the bacterial protein analog to eukaryotic MHC classII molecules, and IgG binding protein, may interfere with host immune defenses [28]. Up to 20 genes encoding hypothetical proteins were up-regulated (30.3%) while four hypothetical proteins were down-regulated (11.1%). Interestingly, a gene encoding the conserved hypothetical protein SAR0996 was the most prominently up-regulated gene (up to 97.3 fold). Moreover, the data revealed that two ORF transcripts of unknown function (SAR0437 and SAR0761) showed large increases in expression (>30 fold) after exposure to rhodomyrtone and may feature in the primary mode of action of the compound. A more detailed examination of these gene responses will be clarified in further studies.

EMRSA-16 genes down-regulated by rhodomyrtone fell into a number of functional categories that included those involved in nucleotide and amino acid metabolism and also membrane and transporter proteins. A phosphor transferase system (PTS) gene, *mtlA*, was expressed at a markedly decreased level (12.44-fold reduction). The gene encoding L-lactate dehydrogenase 1, *ldh*1, was down-regulated more than 15-fold by rhodomyrtone. This gene is involved in pyruvate utilization, converting pyruvate to lactate. A number of genes related to nucleotide metabolism were down-regulated by rhodomyrtone, including DNA gyrase subunit B genes (*gyrB*), DNA polymerase III subunit delta' (*holB*), purine operon repressor (*purR*), transcription antitermination protein (*nusG*), DNA-directed RNA polymerase beta chain protein (*rpoB*), succinate dehydrogenase cytochrome b558 (*sdhC*), ribosomal large subunit pseudouridine synthase B (*rluB*), tRNA-specific 2-thiouridylase Mnm (*mnmA*), queuine tRNA-ribosyltransferase putative methyltransferase (*tgt*), RNA polymerase sigma-B factor (*sigB*), transcription termination factor (*rho*), tRNA (guanine-N(7)-)-methyltransferase (*trmB*) and tRNA pseudouridine synthase A (*truA*).

### Quantitative Reverse Transcription-polymerase Chain Reaction (qRT-PCR)

Fourteen gene-specific primers were used, including (i), ten genes identified by microarray analysis as modulated by rhodomyrtone, *asd*, *dapA*, *kdpF, thrC*, *scdA*, SAR0996, SAR0437, SAR0761, *sigB*, and *rpoB*, and (ii), four genes adopted as negative controls, *lysA*, *ftsH*, *ftsZ*, and *murE*, that were not differentially expressed by rhodomyrtone (Table 1). Gene expression data obtained for *asd*, *dapA*, *thrC*, SAR0996, SAR0437, SAR0761, *sigB*, and *rpoB* were similar for both microarray analysis and qRT-PCR, even though the fold change values of most genes obtained from qRT-PCR were lower than those obtained by microarray analysis.

**Table 1:** Oligonucleotide primers used for qRT-PCR analysis.

Primer target^a^		Nucleotide sequence (5′ to 3′)^b^
*asd*	F	CAAGTCAATGGCGTATGG
	R	GGCACAACAGACTGAATC
*dapA*	F	ACTACTGCTGAGAGCCCTAC
	R	GTTCCAGTTCCAGCTATGAC
*ftsH*	F	TAGAGCGGTTGCAGGTGAAG
	R	ACCACGTTGACGACCAACAG
*ftsZ*	F	TTACTGGTGGCGAGTCATTG
	R	TTTACGCTTGTTCCGAATCC
*kdpF*	F	GAGGCGTTAAGCTATCATGC
	R	CATGGAGCGAATATGGAAGG
*lysA*	F	TTAGACTGCCGGTATCTTGG
	R	CAATAACGTCGCCTGTACTG
*murE*	F	TGATTCACGTACAGCGAGAG
	R	CTTAATGTGTCCGGCACAAC
*rpoB*	F	CGCCTAAAGGTGTAACTGAG
	R	GATAATGTGTCGTCGCCTTC
SAR0996	F	GAAGCGTTTGTCACAACC
	R	ATTGACGAGTGCGTCATC
SAR0437	F	CCGTCTTGCTTAGCTTTG
	R	GAAGCAGATGCGAAAGTG
SAR0761	F	TTAATTTAGCGCCGCCGAAG
	R	TCGCAATGGTTGACTACG
*scdA*	F	AGCAAGGTGAGGTAGTAGAC
	R	AGTACCACACGCTTCTATCG
*sigB*	F	GCCGTTCTTTGAAGTCTG
	R	TATCTGATCGCGAACGAG
*thrC*	F	TTTGGCTTCCAAGCTGAAGG
	R	TCCCAACTAGCAGGATTACC
16s rRNA	F	GACGGTCTTGCTGTCACTTA
	R	AGTTCCAGTGTGGCCGATCA

a Based on the annotation of MRSA252 genome.

b Forward (F) and reverse (R) primers.

**Figure 1:**
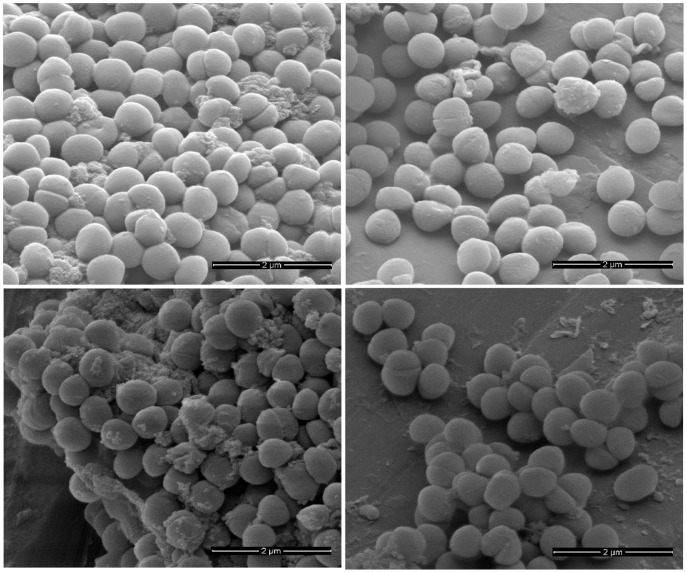
Time course analysis of gene expression in MRSA following exposure to rhodomyrtone. RNA samples were extracted from rhodomyrtone-treated cells (0.5 µg/ml, 0.5MIC) after incubation for 1 h, 4 h, and 18 h. Gene expression levels were normalized to 16s rRNA (*p*-value of ≤0.05).

**Figure 2:**
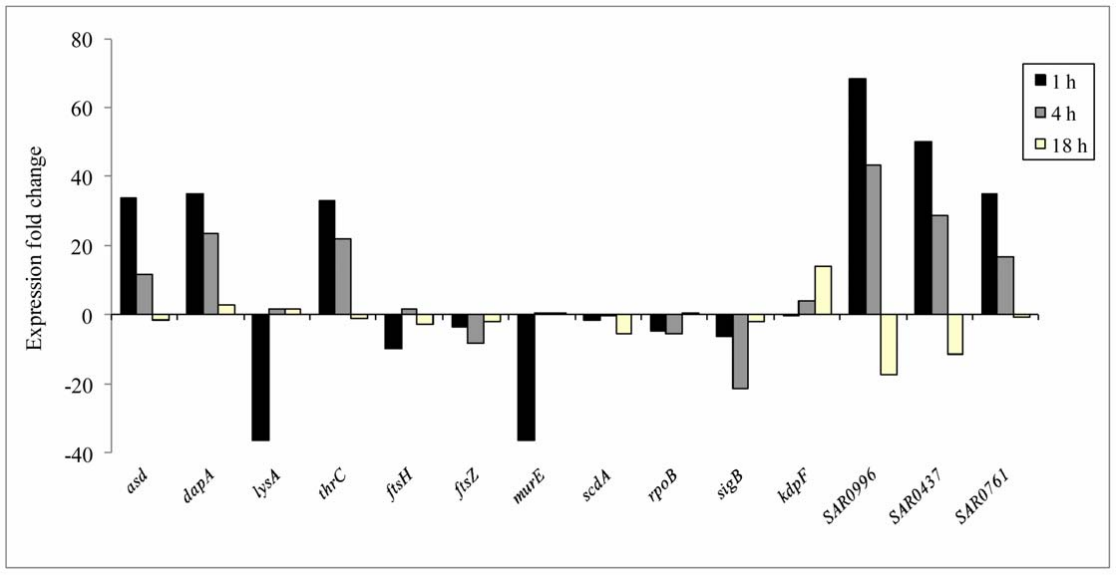
Schematic representation of the diaminopimelate (DAP) pathway in staphylococci. The pathway depicts expression of DAP biosynthesis related genes induced or reduced in rhodomyrtone-treated MRSA after 1 h incubation. Gray boxes: genes that were significantly represented by microarray; pink boxes: genes that were significantly represented by qRT-PCR. Blue arrow and red arrow represented an increase or decrease in expression fold change, respectively.

A time course analysis of 14 genes by qRT-PCR of EMRSA-16 exposed to rhodomyrtone for 1, 4, and 18 h was carried out (Figure 1). After 1 and 4 h exposure, *asd* and *dapA*, involved in the DAP biosynthetic pathway, and the threonine pathway gene *thrC* were up-regulated. After incubation for 18 h, there was no significant change in expression of *asd* and *thrC* while *dapA* continued to be over-expressed but at a lower level than after 1 and 4 h incubation. *lysA*, encoding *meso*-DAP-decarboxylase, was down-regulated at the 1 h timepoint and there was no significant change after incubation for 4 and 18 h. Down-regulation of gene expression after 1 h treatment with rhodomyrtone for 1 h was observed in genes involved in bacterial cell wall biosynthesis and cell division, including *ftsH*, *ftsZ*, *murE*, and *rpoB*; *rpoB* encodes the *β* subunit of RNA polymerase. Mutation of *rpoB* results in repression of urease genes and up- and downregulation of genes involved in cell wall biosymthesis and cell membrane metabolism [29]. *rpoB* remained under-expressed after 4 h exposure to the agent but was digfferentially expressed by 18 h. At 1 h, only *murE* was up-regulated (>19 fold); *ftsZ* and *rpoB* were down-regulated and *ftsZ* and *scdA* were unchanged. *ftsH, ftsZ,* and *scdA* was down-regulated while levels of *murE* RNA were not significantly different after 18 h incubation with rhodomyrtone. Coordinated cell wall metabolism is critical for bacterial cell integrity, playing a significant role in the determination of cell shape and cell division, in resistance to external stress conditions such as osmotic pressure, and the wall is involved in host-pathogen interactions [30]. Microarray data revealed *scdA* to be 5.14-fold up-regulated following exposure to rhodomyrtone at 0.5 µg/ml for 1 h. Scanning electron microscopy (SEM) revealed no abnormalities with respect to morphology, probably reflecting the shorter time intervals for changes in gene expression in comparison to those required for manifestation of phenotypic changes. *scdA* encodes the cell wall biosynthesis protein Scd, which plays an important role in staphylococcal cell division and cell wall turnover. Mutation of this gene has revealed dramatic changes in morphology characterized by aberrant placement of septa [31]. Proteome analysis data from previous studies on a clinical MRSA strain indicated down-regulation of ScdA following rhodomyrtone treatment at 0.174 µg/ml for 18 h [18]. However, qRT-PCR revealed no obvious changes in *scdA* expression. After 1 h incubation, *murE* gene did not appear to be modulated when bacteria were subjected to microarray analysis but was significantly up-regulated when qRT-PCR was employed. *murE* encoding the UDP-N-acetylmuramoyl-L-alanyl-D-glutamate:meso-diaminopimelate ligase and is a essential enzyme in the assembly of the stem peptide of peptidoglycan [32]. The gene encoding the cell division protein FtsZ was not modulated as determined by microarray but was significantly down-regulated 1 h after challenge with rhodomyrtone by qRT-PCR. Figure 2 summarises rhodomyrtone-induced changes, after I h exposure of MRSA, in the expression of genes of the aspartate pathway. *sigB* is key to the regulation of stress responses enabling survival under stresses induced by antibiotics and host bactericidal factors. In this study, *sigB* was down-regulated after treatment at all time-points, indicating responses of the bacteria to environmental stresses.

Genes of unknown function (encoding hypothetical proteins), including SAR0996 (encoding conserved hypothetical protein), SAR0437 (encoding putative exported protein), and SAR0761 (encoding putative lipoprotein), were highly up-regulated at the 1 and 4 h time points. After 18 h, down-regulation of SAR0996 and SAR0437 was evident while SAR0761 showed no change. Such genes may be relevant to the mode of action of rhodomyrtone in MRSA, as substantial changes in expression were observed at all three time points. We will undertake the characterization of these genes in order to shed light on the mechanism of action of the bioactive compound.

### SEM

**Figure 3:**
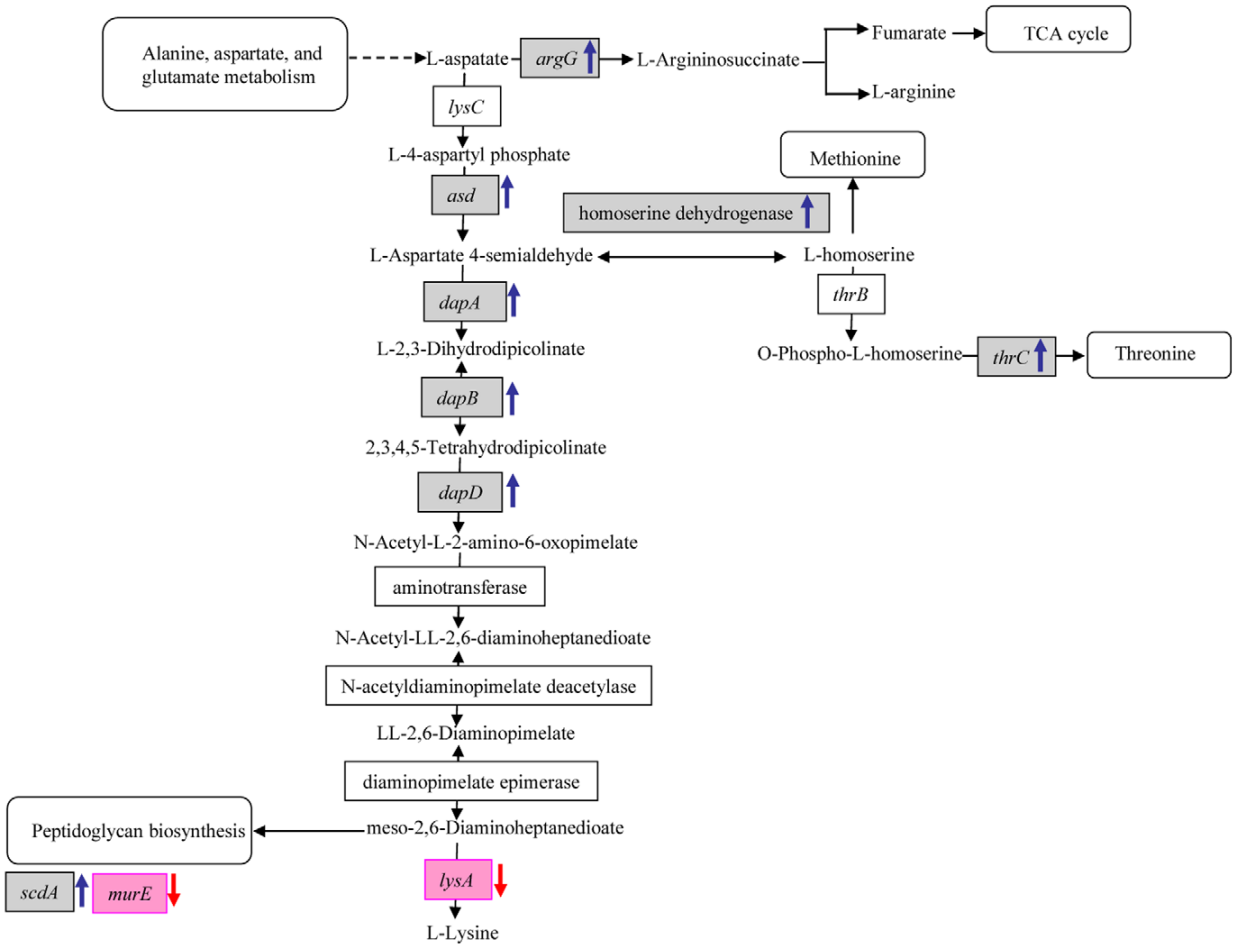
Scanning electron micrographs demonstrate the effect of rhodomyrtone on EMRSA-16 cell morphology. The bacteria were grown in MHB containing 0.5 µg/ml rhodomyrtone (0.5MIC) and incubated for 4 h (A). Untreated control cultures were grown in MHB supplemented with DMSO and incubated for 4 h (B). Scale bars = 2 µm.

EMRSA-16 was incubated for 1 and 4 h with rhodomyrtone at a concentration of 0.5MIC (0.5 µg/ml) and the cells examined by SEM. No obvious differences in bacterial morphology and cell surface architecture between rhodomyrtone-treated cells and the untreated control were observed at either time point (Figure 3A–3B).

### Effects on Amino Acid Composition of Peptidoglycan

To determine if the addition of rhodomyrtone at 0.5MIC caused modification of the murein structure of MRSA in terms of amino acid composition, peptidoglycan from EMRSA-16 cells grown in MHB supplemented with rhodomyrtone for 4 h was purified, analyzed and compared to material extracted from untreated control cells. Cell wall amino acids were quantified by reversed phase high-performance liquid chromatography (HPLC). Molar mass ratios of peptidoglycan-derived amino acids, glycine, alanine, and lysine, were normalized with respect to glutamic acid. No obvious differences in murein amino acid content were identified when rhodomyrtone-treated cells and untreated controls were compared, suggesting that rhodomyrtone did not affect muropeptide composition of the bacterial peptidoglycan (Table 2).

**Table 2:** Analysis of amino acid composition of purified peptidoglycan of EMRSA-16 in the presence or absence of rhodomyrtone.

Molar mass ratio relative to glutamic acid^a^		
Amino acid	Rhodomyrtone-treated cells	Untreated cells
Alanine	1.70	2.13
Glutamic acid	1.00	1.00
Glycine	3.33	3.94
Lysine	0.97	1.05

a Molar ratios normalized to Glutamic acid = 1.0, based on amino acid composition and molecular weight.

In summary, microarray analysis demonstrated that rhodomyrtone induces changes in gene transcription of MRSA. Thus, genes involved in the biosynthesis of amino acids, in particular those of the diaminopimelate biosynthetic pathway, in the biogenesis of the cell envelope, in transporter proteins and in nucleotide metabolism were significantly modulated. Genes encoding the hypothetical proteins SAR0996, SAR0437, and SAR0761 were highly up-regulated and we intend to examine their potential role in the antibacterial action of rhodomytrone.

## Materials and Methods

### Purification of Rhodomyrtone

The purification and structural elucidation of rhodomyrtone, a major bioactive principle from the leaf of *R. tomentosa*, have been previously published by our research group [15], [16]. The purity of rhodomyrtone has been confirmed by nuclear magnetic resonance and mass spectrometry [14], [16]. Rhodomyrtone was dissolved in 100% dimethyl sulphoxide (DMSO) prior to addition to Mueller Hinton broth (MHB, Oxoid). Bacterial cultures serving as untreated controls contained DMSO but not rhodomyrtone.

### Bacterial Strains, Growth Conditions, and Antibacterial Susceptibility

Epidemic MRSA isolate EMRSA-16 was from a clinical sample obtained at the Royal Free Hospital, London, UK [19]. The bacteria were grown without shaking in MHB at 37°C. MIC was determined by broth microdilution method according to Clinical and Laboratory Standards Institute procedures [20]. In brief, twofold serial dilutions of rhodomyrtone in a 96-well microtiter plate were prepared in MHB to obtain a concentration range of 0.0625 to 128 µg/ml. Exponential phase bacteria were added to each well to give an inoculum of 5×10^5^ cfu/ml in a total volume of 200 µl. After incubation at 37°C for 16–18 h, the extent of inhibition of bacterial growth was determined using a spectrophotometer against a blank well (MHB containing 1% DMSO). MIC was defined as the lowest concentration of the agent that completely inhibited bacterial growth. For each assay, three independent experiments were performed in triplicate.

### Preparation of RNA

Total RNA was extracted using FastRNA® Pro Blue kit (Q-biogen, UK) according to the manufacturer’s instruction. For microarray analysis, an overnight culture of EMRSA-16 was added to fresh MHB to provide an initial inoculum of approximately 10^6^ cfu/ml. Bacterial cultures were incubated at 37°C to exponential phase (optical density at 600_nm_ = 0.15). Rhodomyrtone was added to the cultures to give a final concentration of 0.5 µg/ml. The same concentration of DMSO used in rhodomyrtone-treated cells was added to the untreated control. The cultures were incubated at 37°C for 1, 4, or 18 h. The bacterial pellets of each time point were collected by centrifugation at 8,000×g for 30 min and washed twice with phosphate buffer saline (PBS). A double volume of RNAprotect Bacteria Reagent (QIAGEN, UK) was added to the pellets. The mixture was incubated at room temperature for 5 min and the bacterial cells centrifuged (3,500×g; 4°C; 10 min). Total RNA was extracted from the bacteria using the manufacturer’s “Fast prep” protocol for the RNeasy Mini-Prep Kit (QIAGEN, UK). The bacterial DNA was removed by DNase digestion steps with RNase-free DNase (QIAGEN, UK) and RNA further purified using RNeasy Mini protocol. RNA quality and quantity was determined by agarose gel electrophoresis, NanoDrop 2000 spectrophotometer (Nanodrop Technologies), and Bioanalyzer 2100 (Agilent Technologies). All samples were stored at -80°C until required.

### Microarray and Data Analysis

The microarray analysis used in this study has been previously described by Doyle *et. al.* [21]. The array contains PCR products representing all predicted open reading frames from the initial seven *S. aureus* genome sequencing projects [22]. The array design is available in BµG@Sbase (Accession No. A-BUGS-17; http://bugs.sgul.ac.uk/A-BUGS-17) and also ArrayExpress (Accession No. A-BUGS-17). RNA isolated from bacteria exposed to rhodomyrtone for 1 h was transcribed into cDNA and concomitantly labeled by incorporation of Cy3 and Cy5 (GE Healthcare) using superscript III reverse transcriptase (Invitrogen, UK). cDNA was purified using MinElute kit (QIAGEN, UK), the probes pooled, hybridized overnight to the *S. aureus* microarray at 65°C and subjected to stringent washing. Hybridization data was analyzed by Affymetrix 428 scanner and quantified using Bluefuse for Microarrays 3.5 software (BlueGnome). Data analysis was performed in GeneSpring GX 7.3 (Agilent Technologies) by median-normalized Cy5/Cy3 ratio intensity for three biological replicates. Only genes whose expression ratio showed at least two-fold difference with Benjamini and Hochberg false discovery rate ≤0.05% in the presence of rhodomyrtone were regarded as being significantly different from the control. Fully annotated microarray data have been deposited in BµG@Sbase (accession number E-BUGS-136; http://bugs.

sgul.ac.uk/E-BUGS-136) and also ArrayExpress (accession number E-BUGS-136). Microarray analysis data were further validated by qRT-PCR.

### qRT-PCR

The reaction was performed on cDNA generated from RNA samples collected after 1, 4, or 18 h incubation. qRT-PCR was carried out using Brilliant II SYBR® Green qRT-PCR one-step kit (Stratagene). The reaction was run in Rotor gene 3000 (Corbett Research) using the primers listed in Table 1. The condition applied for PCR was 95°C for 10 min, followed by 55 cycles of 95°C for 30 s, 57°C for 30 s, and 72°C for 30 s. The comparative threshold method was used to determine relative quantification of mRNA abundance. Changes in mRNA expression level were calculated after normalization to the internal control, 16S rRNA bacterial house-keeping gene.

### SEM

EMRSA-16 cells were collected by centrifugation (8,000×g at 4°C for 5 min) following exposure to rhodomyrtone at a concentration of 0.5 µg/ml for 1 or 4 h. The cells were washed twice with PBS and fixed in 1.5% glutaraldehyde for at least 2 h at room temperature. Samples were washed once with 70% ethanol, twice with 100% ethanol, air dried, and adhered to a SEM stub using double sided carbon adhesive discs (Taab Laboratories). The samples were gold-coated using a Q150T Sputter Coater (Quorum Technologies) and viewed with a FEI Quanta 200 FEG Scanning Electron Microscope and an accelerating voltage of 5 kV.

### Isolation of Peptidoglycan

Peptidoglycan was extracted and purified by a modification of the method described by Stranden and coworkers [23]. Briefly, logarithmic phase bacteria were inoculated into MHB containing 0.5 µg/ml of rhodomyrtone and incubated for 4 h at 37°C. The cells were rapidly chilled, harvested by centrifugation (10,000×g; 4°C; 20 min) and washed with 0.01 M PBS. The bacterial cells were broken with glass beads (0.2 mm) using a cell homogenizer (FastPREP FP120, Savant) at a speed setting of 6.0 for 45 s. The samples were placed on ice for 2 min and centrifuged at 14,100×g for 5 min. After removal of the supernatant, the cell pellet was washed with 2 M NaCl. The cell suspension was further washed with 0.5% SDS and incubated at 60°C for 30 min with stirring to remove noncovalently bound components. The bacterial cell wall was isolated by centrifugation at 5,100×g at 25°C for 15 min and washed with water. Protein A was removed by incubation with 0.2 mg of trypsin per ml in 1 M Tris-HCl (pH 7.2) at 37°C for 24 h. The samples were centrifuged, washed several times with buffer and water, and then resuspended in 1 ml of 10% (w/vo) aqueous trichloroacetic acid at 4°C for 18 h to remove teichoic acid. Purified murein was isolated by centrifugation at 14,100×g for 5 min, washed with water, and lyophilized using Freeze Dryer (ALPHA 1–2 LDplus, Christ).

### Peptidoglycan Amino Acid Analysis

Determination of the amino acid content of peptidoglycan was preformed by Alta Bioscience (University of Birmingham, UK). The lyophilized, isolated peptidoglycan was hydrolyzed with 5.8M HCl under vacuum at 110°C for 24 h. Amino acids were analyzed in an amino acid analyzer based on Waters HPLC hardware with sodium citrate buffer system. The amino acids were detected using ninhydrine reagent.

## Supporting Information

Table S1 **Genes up-regulated in rhodomyrtone-treated EMRSA-16.**
(DOC)

Table S2 **Genes down-regulated in rhodomyrtone-treated EMRSA-16.**
(DOC)
