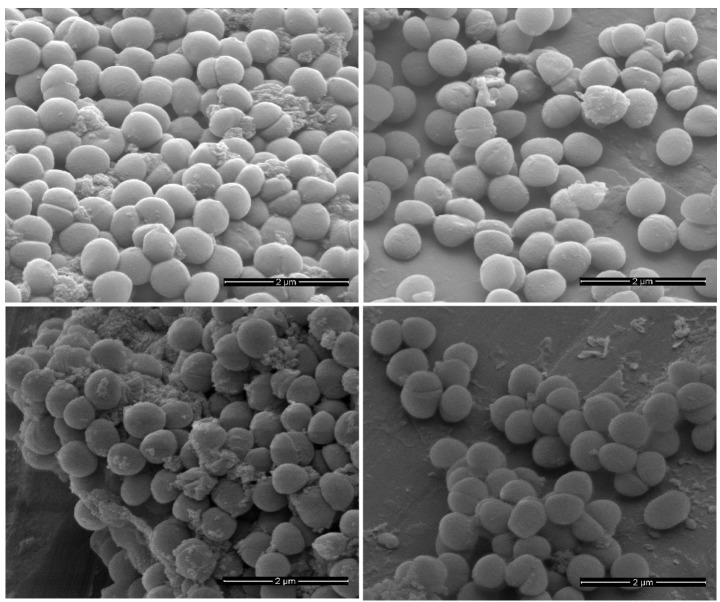# Correction: Transcriptome Analysis of Responses to Rhodomyrtone in Methicillin-Resistant *Staphylococcus aureus*

**DOI:** 10.1371/annotation/621cdbb7-0918-4ec4-8fa0-83ef4d97a344

**Published:** 2013-07-12

**Authors:** Wipawadee Sianglum, Potjanee Srimanote, Peter W. Taylor, Helena Rosado, Supayang P. Voravuthikunchai

The images for Figures 1, 2, and 3 were misplaced. The legends for the figures are placed correctly.

The correct Figure 1 can be found here: 

**Figure:**
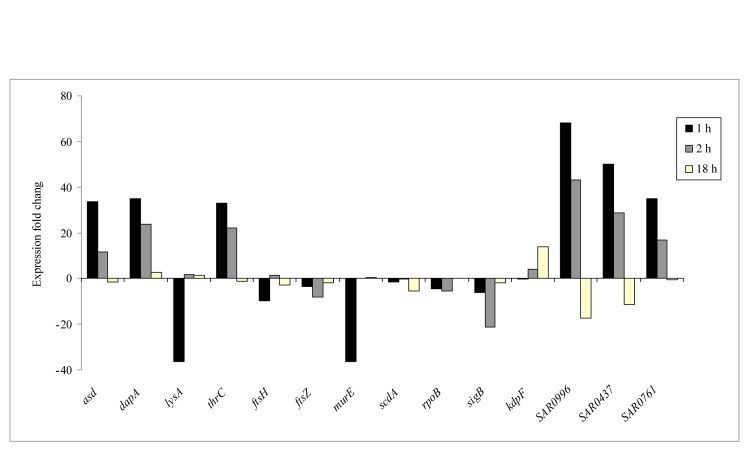


The correct Figure 2 can be found here: 

**Figure:**
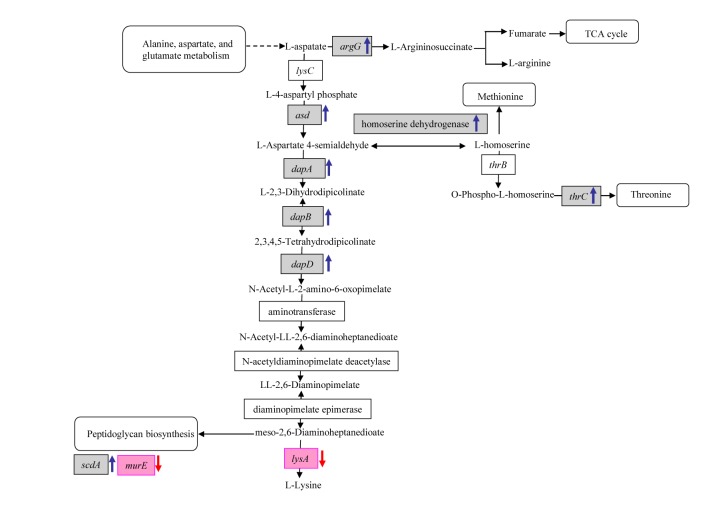


The correct Figure 3 can be found here: 

**Figure:**