# Supplementary material for: Transcriptome Analysis of Responses to Rhodomyrtone in Methicillin-Resistant Staphylococcus aureus
Source: PLoS One. 2012 Sep 27;7(9):e45744. doi: 10.1371/journal.pone.0045744 (PMC3459976; doi:10.1371/journal.pone.0045744)
Supplement: Table S1 — Genes up-regulated in rhodomyrtone-treated EMRSA-16. (DOC) [file pone.0045744.s001.doc]

**Table S1.** Genes up-regulated in rhodomyrtone-treated EMRSA-16

| Category and locus | Gene | Gene product description | Expression-fold |
| --- | --- | --- | --- |
|  |  |  | change |
| **Amino acid metabolism** |  |  |  |
| SAR0923 | *argG* | argininosuccinate synthase | 7.35 |
| SAR1338 | *dhoM* | homoserine dehydrogenase | 34.65 |
| SAR1339 | *thrC* | threonine synthase | 19.39 |
| SAR1406 | *asd* | aspartate semialdehyde dehydrogenase | 64.84 |
| SAR1407 | *dapA* | dihydrodipicolinate synthase | 85.49 |
| SAR1408 | *dapB* | dihydrodipicolinate reductase | 88.30 |
| SAR1409 | *dapD* | tetrahydrodipicolinate | 51.45 |
|  |  | acetyltransferase |  |
| **Cell wall metabolism** |  |  |  |
| SAR0256 | *scdA* | cell wall metabolism protein | 5.14 |
| **Exported protein** |  |  |  |
| SAR0437 | *­* | putative exported protein | 58.93 |
| SAR1094 | *­* | putative exported protein | 2.25 |
| SAR2295 | *­* | putative exported protein | 7.49 |
| SAR2615 | *­* | putative exported protein | 5.35 |
| **Hypothetical protein** |  |  |  |
| lpl5 | *lpl5* | hypothetical protein | 2.99 |
| MW0754 | *­* | hypothetical protein | 2.10 |
| SACOL1336 | *­* | hypothetical protein | 2.27 |
| SACOL2406 | *­* | hypothetical protein | 3.67 |
| SACOL2454 | *­* | hypothetical protein | 3.05 |
| SAOUHSC_01090 | *­* | hypothetical protein | 2.10 |
| SAR0090 | *­* | hypothetical protein | 2.50 |
| SAR0421 | *­* | conserved hypothetical protein | 9.90 |
| SAR0448 | *­* | hypothetical protein | 2.05 |
| SAR0996 | *­* | conserved hypothetical protein | 97.32 |
| SAR1342 | *­* | hypothetical protein | 4.14 |
| SAR1415 | *­* | hypothetical protein | 3.02 |
| SAR1572 | *­* | conserved hypothetical protein | 3.71 |
| SAR1682 | *­* | hypothetical phage protein | 2.09 |
|  |  | (pseudogene) |  |
| SAR2097 | *­* | hypothetical phage protein | 3.02 |
| SAR2098 | *­* | hypothetical phage protein | 2.53 |
| SAR2228 | *­* | conserved hypothetical protein | 3.71 |
| SAR2380 | *­* | hypothetical protein | 2.12 |
| SAR2561 | *­* | conserved hypothetical protein | 6.88 |
| SAR2617 | *­* | hypothetical protein | 3.45 |
| **Lipoprotein** |  |  |  |
| SAR0445 | *­* | putative lipoprotein | 2.66 |
| SAR0761 | *­* | putative lipoprotein | 31.89 |
| SAR0872 | *­* | putative lipoprotein | 10.68 |
| SAR0997 | *­* | putative lipoate-protein ligase A | 2.72 |
| SAR1402 | *­* | phosphate-binding lipoprotein | 13.74 |
| **Membrane protein** |  |  |  |
| SAR0128 | *­* | putative membrane protein | 4.45 |
| SAR0420 | *­* | putative membrane protein | 5.20 |
| SAR0442 | *­* | putative membrane protein | 3.18 |
| SAR0760 | *­* | putative membrane protein | 8.79 |
| SAR2451 | *­* | putative membrane protein | 3.11 |
| SAR2613 | *­* | putative membrane protein | 15.76 |
| SAR2783 | *­* | putative membrane protein | 2.88 |
| **Nucleotide metabolism** |  |  |  |
| SAR2372 | *ureA* | urease gamma subunit | 3.90 |
| SAR2374 | *ureC* | urease alpha subunit | 2.74 |
| **Transport protein** |  |  |  |
| SACOL2069 | *kdpF* | K+-transporting ATPase, F subunit | 4.08 |
| SAR0870 | *­* | ABC transporter ATP-binding | 9.51 |
|  |  | protein |  |
| SAR0871 | *­* | ABC transporter permease protein | 8.73 |
| SAR1079 | *­* | manganese transport protein MntH | 5.34 |
| SAR2701 | *­* | ABC transporter ATP-binding | 3.67 |
|  |  | protein |  |
| SAR2781 | *vraD* | ABC transporter ATP-binding | 11.01 |
|  |  | protein |  |
| **Virulence factor** |  |  |  |
| SAR2030 | *­* | MHC class II analog | 27.82 |
| SAR2508 | *sbi* | IgG-binding protein | 3.24 |
| **Others** |  |  |  |
| SAR1280 | *­* | glutathione peroxidase | 2.39 |
| SAR1410 | *­* | putative peptidase | 6.38 |
| SAR2121 | *­* | putative carbon-nitrogen hydrolase | 8.15 |
| SAR2290 | *­* | aldo/keto reductase family protein | 4.04 |
| SAR2522 | *­* | putative glycerate kinase | 6.32 |
| SAR2628 | *clpL* | putative ATP-dependent protease | 7.56 |
|  |  | ATP-binding subunit ClpL |  |
| SAR2661 | *­* | putative hydrolase | 5.54 |
| SAR2708 | *­* | putative esterase | 2.91 |
| SAR2779 | *­* | putative N-acetyltransferase | 3.58 |
| SAR2784 | *­* | transposase (pseudogene) | 6.50 |
|  |  |  |  |
|  |  |  |  |
|  |  |  |  |
