# Supplementary material for: Transcriptome Analysis of Responses to Rhodomyrtone in Methicillin-Resistant Staphylococcus aureus
Source: PLoS One. 2012 Sep 27;7(9):e45744. doi: 10.1371/journal.pone.0045744 (PMC3459976; doi:10.1371/journal.pone.0045744)
Supplement: Table S2 — Genes down-regulated in rhodomyrtone-treated EMRSA-16. (DOC) [file pone.0045744.s002.doc]

| **Table S2.** Genes down-regulated in rhodomyrtone-treated EMRSA-16 | | | |
| --- | --- | --- | --- |
| EMRSA-16 | Gene | Gene Product Description | Expression-fold  change |
|
| **Amino acid metabolism** |  |  |  |
| SAR0459 | *cysM* | pyridoxal-phosphate dependent | ­3.87 |
|  |  | enzyme |  |
| SAR0589 | *­* | putative amino acid permease | ­4.55 |
| SAR2400 | *­* | putative amino acid permease | ­2.85 |
| **Carbohydrate metabolism** |  |  |  |
| SAR0234 | *ldh1* | L-lactate dehydrogenase 1 | ­15.35 |
| **Hypothetical protein** |  |  |  |
| SAR1834 | *­* | conserved hypothetical protein | ­3.63 |
| SAR2243 | *­* | hypothetical protein | ­2.94 |
| SAR2569 | *­* | hypothetical protein | ­2.86 |
| **Membrane protein** |  |  |  |
| SAR1051 | *­* | putative membrane protein | ­4.26 |
| SAR1091 | *­* | putative membrane protein | ­3.44 |
| SAR1493 | *­* | putative membrane protein | ­2.44 |
| SAR2179 | *­* | putative membrane protein | ­8.96 |
| SAR2647 | *­* | putative membrane protein | ­2.83 |
| **Nucleotide metabolism** |  |  |  |
| SAR0005 | *gyrB* | DNA gyrase subunit B | ­2.41 |
| SAR0485 | *holB* | DNA polymerase III, delta' | ­2.23 |
|  |  | subunit |  |
| SAR0497 | *purR* | pur operon repressor | ­2.10 |
| SAR0540 | *nusG* | transcription antitermination protein | ­2.55 |
| SAR0547 | *rpoB* | DNA-directed RNA polymerase beta |  |
|  |  | chain protein | ­5.48 |
| SAR1120 | *sdhC* | succinate dehydrogenase |  |
|  |  | cytochrome b558 | ­3.65 |
| SAR1569 | *rluB* | ribosomal large subunit |  |
|  |  | pseudouridine synthase B | ­2.05 |
| SAR1701 | *mnmA* | tRNA-specific 2-thiouridylase MnmA | ­2.61 |
| SAR1719 | *tgt* | queuine tRNA-ribosyltransferase | ­3.15 |
| SAR1833 | *trmB* | tRNA (guanine-N(7)-)-methyltransferase | ­3.71 |
| SAR2152 | *sigB* | RNA polymerase sigma-B factor | ­5.87 |
| SAR2209 | *rho* | transcription termination factor | ­2.35 |
| SAR2302 | *truA* | tRNA pseudouridine synthase A | ­2.74 |
| **Phosphotransferase system** | |  |  |
| SAR2244 | *mtlA* | PTS system, mannitol-specific IIBC | ­12.44 |
|  |  | component |  |
| **Transporter protien** |  |  |  |
| SAR0648 | *tagG* | teichoic acid ABC transporter | ­2.55 |
|  |  | Permease protein |  |
| SAR1049 | *­* | cobalt transport protein | ­3.86 |
| SAR1050 | *­* | ABC transporter ATP-binding | ­5.79 |
|  |  | protein |  |
| **Virulence factor** |  |  |  |
| SAR0842 | *clfA* | clumping factor | ­4.53 |
| **Others** |  |  |  |
| SAR0033 | *knt* | kanamycin nucleotidyltransferase | ­6.94 |
| SAR0911 | *mnhD* | Na+/H+ antiporter subunit | ­2.21 |
| SAR1018 | *­* | putative hydrolase | ­2.27 |
| SAR1857 | *­* | putative exported protein | ­3.29 |
| SAR0644 | *sirR* | putative metalloregulator | ­2.25 |
|  |  |  |  |
|  |  |  |  |
|  |  |  |  |
